# Data-Driven Prediction and Inverse Design of Fluoride Glasses via Explainable GA-BP Neural Networks

**DOI:** 10.3390/ma19091685

**Published:** 2026-04-22

**Authors:** Runze Zhou, Xinqiang Yuan, Longfei Zhang, Chi Zhang, Hongxing Dong, Long Zhang

**Affiliations:** 1 School of Physics and Optoelectronic Engineering, Hangzhou Institute for Advanced Study, University of Chinese Academy of Sciences, Hangzhou 310024, China; 2 Research Center of Infrared Optical Materials, Key Laboratory of High Power Laser Materials, Shanghai Institute of Optics and Fine Mechanics, Chinese Academy of Sciences, Shanghai 201800, China

**Keywords:** fluoride glass, neural network, refractive index, density, inverse design, SHapley Additive exPlanations

## Abstract

**Highlights:**

1. Data-driven framework for fluoride-glass prediction and inverse design.
2. The genetic-algorithm–optimized backpropagation neural network jointly predicts refractive index and density.
3. Bayesian hyperparameter tuning enhances accuracy and robustness.
4. Constraint-aware inverse design ranks feasible compositions by targets & similarity.
5. SHapley Additive exPlanations analysis is employed to quantify feature importance and enhance the interpretability of the model.

**Abstract:**

With the increasing application of novel glass materials in the field of optics, traditional empirical and trial-and-error approaches to glass development are gradually becoming insufficient to meet escalating performance demands. In this study, we propose a neural network-based machine learning method for the design of advanced fluoride glass materials. Predictive models for density and refractive index were first developed based on online fluoride glass datasets. Moreover, SHapley Additive exPlanations (SHAP) analysis was adopted to uncover the quantitative composition-property relationship. Then, the well-trained model was employed for inverse design, identifying specific compositions that fulfill desired properties in terms of density and refractive index. Finally, several recommended compositions were experimentally validated and the measured density and refractive index matched well with the corresponding input values, thereby confirming the effectiveness of the proposed method in designing new fluoride glass materials.

## 1. Introduction

Modern optical glasses are typically engineered through the formulation of various oxides and specific additives to meet specific performance requirements across diverse applications. In recent years, fluoride glasses have attracted significant research interest owing to their distinct material properties [1,2,3,4,5,6]. In contrast to conventional oxide-based glasses, fluoride glasses exhibit a broader optical transmission window in the range from 0.25 to 8 μm, a lower refractive index with reduced dispersion, and an exceptionally low thermo-optic coefficient. These unique properties render them particularly promising for mid-infrared applications including infrared optical fibers, high-power laser windows, missile domes, and multispectral imaging systems [7,8]. However, at present the development of fluoride glasses remains heavily reliant on traditional trial and error methods, which is time intensive and laborious [9]. Moreover, fluoride glasses not only have complex compositions and broad chemical variability, but also are highly sensitive to minor composition changes, which pose great challenges for their efficient design and optimization.

Since the launch of the U.S. Materials Genome Initiative (MGI) in 2011, machine learning has been widely adopted in material science and greatly accelerated the development of the new materials [10,11,12,13]. For example, Rao et al. developed an active learning framework that combines machine learning, physical modeling, and experiments to discover high-entropy Invar alloys [11]. Shu Chen et al. employed an unsupervised machine learning framework combined with molecular descriptors and hierarchical clustering to screen organic lithium salts for external Li supply [12]. In the field of glass research, Suresh Bishnoi et al. employed Gaussian process regression and neural networks to predict the Young’s modulus of silicate glasses from sparse datasets, achieving test-set coefficient of determination of 0.98 with Gaussian process regression while simultaneously providing quantitative uncertainty estimates for each prediction [14]. Onbasli et al. developed data-driven glass design models based on neural networks and genetic algorithms, achieving liquidus temperature prediction with an RMSE of 23 °C, which was reduced to 14 °C through cluster-based modeling, and Young’s modulus prediction with a genetic algorithm reaching an RMSE of 1.11 GPa, enabling accurate industrial-scale design of high-strength Gorilla^®^ Glass [15]. Krishnan et al. applied machine learning to predict the dissolution kinetics of silicate glasses, achieving high predictive accuracy with artificial neural networks, with a test-set coefficient of determination of 0.982 and a mean squared error of 0.027, while physics-informed modeling improved extrapolation to untrained compositions [16].

However, despite the broad adoption of machine learning in glass science, existing efforts are still overwhelmingly centered on oxide glasses, whereas studies on fluoride glasses remain rare, despite their technological importance, particularly in optical applications. So far, data-driven property modeling for fluoride compositions is still at an early stage. Additionally, the limited interpretability of machine learning models in glass research remains a major limitation. Glasses are intrinsically multi-component systems in which composition-property relationships are highly nonlinear, strongly coupled, and dominated by interaction effects among constituents [17,18,19,20,21,22,23]. These features not only challenge predictive robustness across high-dimensional composition spaces, but also impede the extraction of physically meaningful, actionable insights from black-box models. In recent years, explainable artificial intelligence methods have gained traction across diverse domains. For instance, Rezazadeh et al. proposed a prototype-attention domain adaptation framework for explainable bearing fault diagnosis, demonstrating that interpretability can be embedded into the model architecture [24]. In the glass domain, Zaki et al. employed SHAP to interpret composition-property relationships in glasses [22]. Nevertheless, such efforts remain largely confined to post hoc analysis, focus on forward modeling or localized optimization, and the resulting explanations often remain insufficient to provide direct, operational guidance for composition exploration and formulation design in highly complex multi-component systems. Therefore, this limitation motivates the need for systematic inverse design, i.e., generating feasible glass compositions that meet target property profiles. However, generalizable inverse-design frameworks for complex, multi-component glasses remain scarce [21,23].

In view of these limitations, this study proposes an integrated framework for predicting and inversely designing the refractive index and density of fluoride glasses: (i) a GA-initialized, Bayesian-optimized neural network for joint density and refractive index prediction; (ii) SHAP-based interpretability analysis to quantify the contribution of individual compositional features; and (iii) constraint-aware inverse design with experimental validation of all generated candidates.

## 2. Methods

### 2.1. Framework Overview

**Figure 1:**
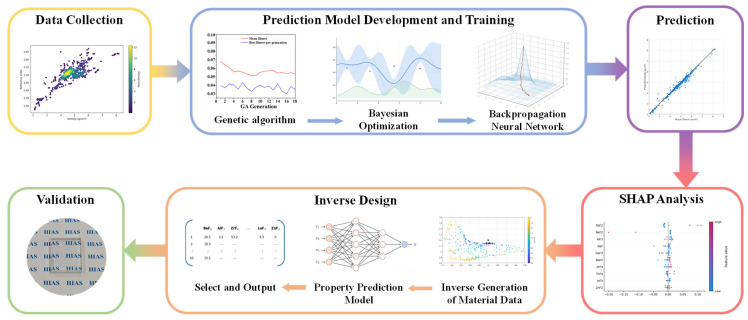
Flow chart of the proposed data-driven framework for fluoride glass property prediction, analysis and inverse design.

Figure 1 illustrates the overall workflow of the proposed computational program, including dataset construction, model training, prediction, inverse design, and validation. Our aim is to establish a reliable, cost-effective, and high-efficiency tool for fluoride glass design.

### 2.2. Data Collection and Preprocessing

All data used in this study were collected from the SciGlass Next database (https://sciglass.uni-jena.de (accessed on 1 March 2026)), which compiles glass properties and corresponding chemical compositions reported in scientific articles, books, and patents. We extracted a dataset consisting of 883 fluoride glass entries, each characterized by density and refractive index ($n_{d}$), with compositions involving 53 different glass-forming components.

**Figure 2:**
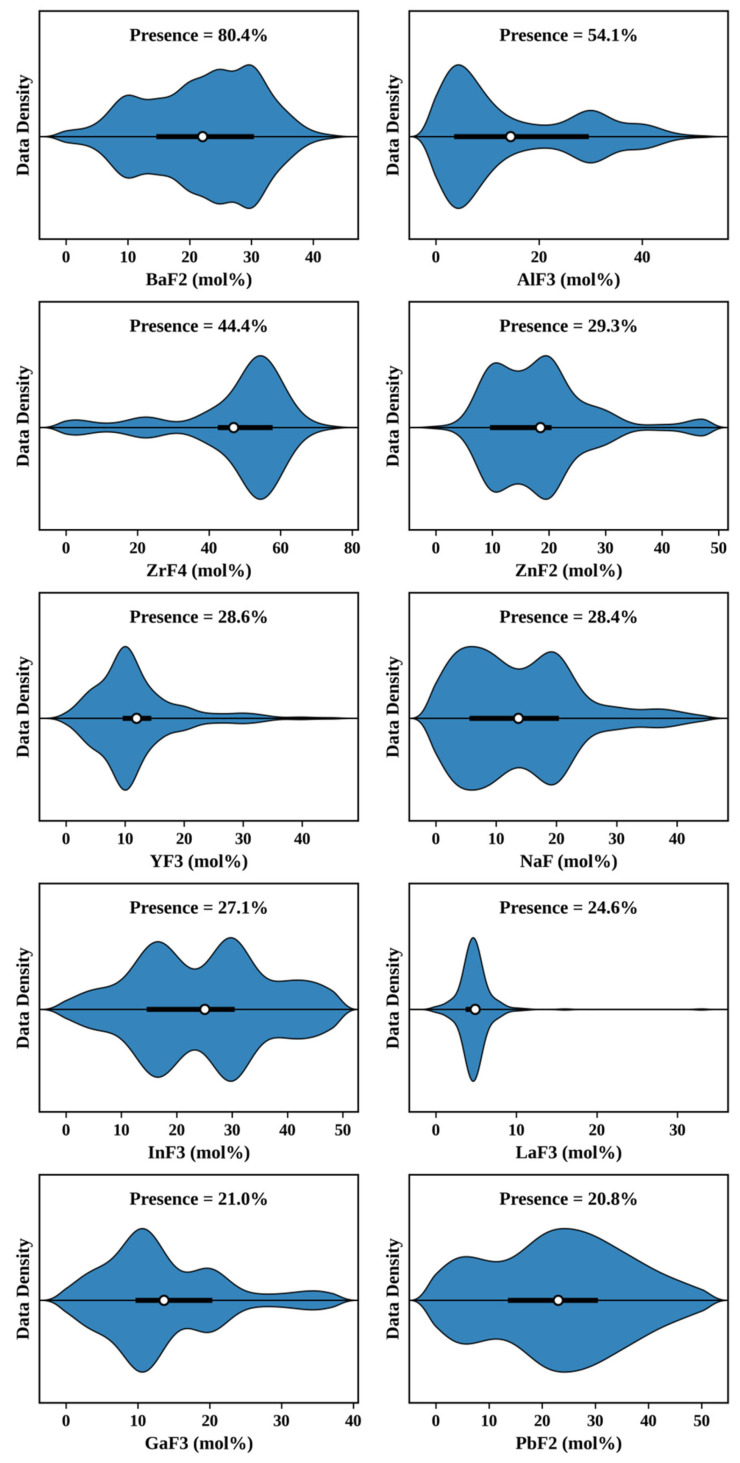
Distributions of non-zero molar fractions for each fluoride component. Only the top ten components with the highest presence in the dataset are shown. The thick segment represents the interquartile range (25–75%), and the white circle denotes the mean value. The presence percentage above each panel reflects the fraction of samples containing the corresponding component.

Figure 2 summarizes the compositional characteristics of the ten most frequently occurring fluoride components in the dataset, reflecting both their molar-fraction ranges and occurrence frequencies. Major constituents such as BaF_2_, AlF_3_, and ZrF_4_ span relatively wide composition ranges, consistent with their roles as key network formers or modifiers in common fluoride-glass families. Other components are distributed within more limited composition intervals, indicating their contributions primarily to structural adjustment and property optimization. Collectively, the broad coverage and heterogeneous distribution of these components demonstrate that the dataset encompasses chemically diverse fluoride-glass systems, providing a representative and sufficiently rich compositional space for machine-learning model training.

**Figure 3:**
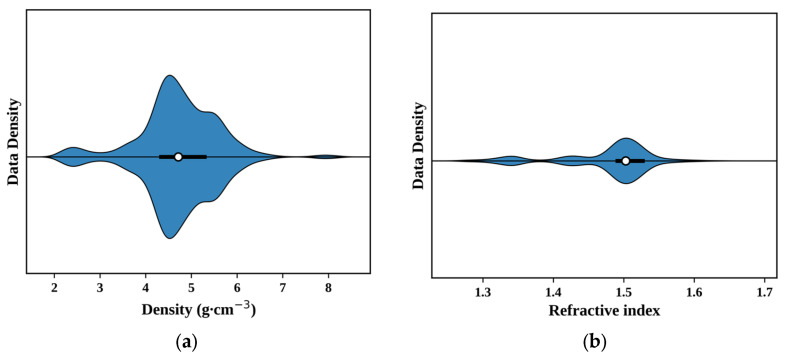
Distributions of (**a**) density and (**b**) refractive index for the fluoride glass samples collected from the SciGlass Next database. The thick segment represents the interquartile range (25–75%), and the white circle denotes the mean value.

Figure 3 presents the statistical distributions of density and refractive index for all fluoride glass samples, highlighting the overall structure and dispersion characteristics of these two key physical properties within the dataset. The pairwise correlations among the top ten features, as well as their correlations with density and refractive index, are summarized in Figure S4.

Before model training, all compositions were normalized to ensure that the total molar percentage equaled 100%. Samples with small deviations (±2%) were proportionally rescaled, whereas samples with larger inconsistencies were excluded. Missing components were assigned a value of zero, consistent with practices in previous glass informatics studies. Density and refractive index were independently normalized to the [0, 1] range using min–max scaling. This preprocessing ensured numerical stability and consistent feature representation across different glasses. The Pearson correlation matrix of all compositional features (Figure S3 in the Supporting Information) confirms that most feature pairs exhibit weak correlations (|r| < 0.3), indicating limited linear multicollinearity.

### 2.3. GA-BP Neural Networks

Neural networks are among the most commonly used machine learning algorithms in glass materials research due to their strong capability in capturing complex nonlinear patterns within data [16,19,21,25,26,27,28,29,30]. In this study, we propose a comprehensive framework that integrates a backpropagation neural network (BPNN), a genetic algorithm (GA), and Bayesian optimization (BO) to systematically predict the density and refractive index of fluoride glasses.

**Figure 4:**
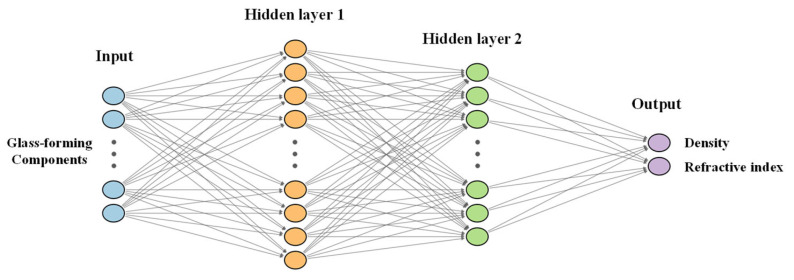
Structure of the constructed BP neural network.

The core predictive model is a feedforward neural network with two hidden layers, trained using a two-stage optimization strategy. In the first stage, the Adam optimizer is employed with a Cosine Annealing warm-restart scheduler for broad exploration of the loss landscape; in the second stage, the L-BFGS algorithm is applied for high-precision parameter convergence [31]. To mitigate the influence of experimental outliers, the Huber (Smooth L1) loss is adopted as the objective function. During training, the network iteratively adjusts its weights and biases to minimize the prediction error, effectively capturing the complex and highly coupled composition–property relationships in multi-component fluoride glasses. The structure of the BPNN is shown in Figure 4.

To reduce the sensitivity of the BPNN to random weight initialization, a genetic algorithm was employed to optimize the initial weights and biases prior to gradient-based training. In the GA, each individual encodes a candidate set of network parameters; through iterative selection, crossover, and mutation operations, the population progressively evolves toward solutions with improved fitness, thereby providing favorable starting conditions that facilitate faster convergence and improved generalization [20].

Building on the GA-initialized network, Bayesian optimization was further adopted to efficiently determine the optimal hyperparameters, including the number of hidden neurons, learning rate, and training epochs. Specifically, Bayesian optimization was implemented using the Optuna framework with a Tree-structured Parzen Estimator sampler to model the mapping from hyperparameter configurations to the validation loss. A total of 50 trials were conducted over a search space covering hidden layer widths, dropout rate, learning rate, and training epochs. By coupling BO with GA-based weight initialization and BP training, the proposed framework achieves stable convergence and improved generalization performance.

A complete summary of the GA, BO, and training configuration parameters is provided in Table S1 of the Supporting Information.

### 2.4. Baseline Models

To quantitatively assess the incremental benefit of each component in the proposed GA-BO-BP framework, we conducted both a systematic benchmarking comparison against four widely used regression models and an ablation study. The following baseline models were trained and evaluated on the identical data splits and evaluation metrics:

(1) Standard BPNN: A two-hidden-layer feedforward neural network with identical architecture but random initialization and manually tuned hyperparameters.
(2) Random Forest (RF): An ensemble of 500 decision trees with default scikit-learn settings, optimized via grid search over max_depth and min_samples_leaf.
(3) XGBoost: Gradient-boosted regression trees with hyperparameters optimized via 5-fold cross-validated grid search over learning rate, max_depth, and n_estimators.
(4) Support Vector Regression (SVR): RBF-kernel SVR optimized via grid search.

All models used the same training, validation, and test partitions, and identical feature preprocessing.

### 2.5. Cross-Validation Strategy

To ensure the robustness and generalizability of the reported results, we employed a 10-fold stratified cross-validation protocol. A fixed test set (10% of the full dataset) was first held out and never used during any training or hyperparameter selection. The remaining 90% of the data was then partitioned into 10 folds stratified by density quantiles, ensuring that each fold reflects the property distribution of the full dataset. For each fold, the model was trained on 9 folds and evaluated on the remaining fold; scalers were re-fitted per fold to prevent any information leakage. The mean and standard deviation of R^2^, MAE, and MSE across all 10 folds are reported to provide confidence intervals for model performance.

To address the concern that compositionally neighboring glasses may appear in both training and test sets under random splitting—potentially overstating extrapolative performance—we additionally performed a family-resolved validation. Samples were grouped by their dominant glass former to evaluate the model’s predictive consistency across distinct chemical regimes.

### 2.6. Inverse Design Formulation

Inverse design aims to identify glass compositions that achieve predefined target properties, such as density and refractive index. Owing to the nonlinearity and non-uniqueness of the composition–property relationship, direct analytical inversion is generally infeasible. In this work, inverse design was formulated as a constrained optimization problem guided by the trained neural network model.

As schematically illustrated in Figure 1, the inverse design workflow follows a sequential procedure. It begins by specifying the target properties, after which a large number of candidate compositions are randomly generated within the allowable compositional domain using a Dirichlet distribution to ensure valid simplex sampling. Each composition is constrained to satisfy: (i) non-negativity of all components, (ii) summation to 100 mol%, and (iii) inclusion of only the user-selected subset of fluoride components. Each candidate composition is then converted into a numerical feature vector and evaluated using the trained neural network model.

A composite scoring function S(x) is employed to rank all candidates:

$$Score=\omega_{\rho }∥\hat{\rho }-\rho_{target}∥+\omega_{n}∥\hat{n}-n_{target}∥+\lambda D_{euclidean}+\varepsilon$$

where $\hat{\rho },\hat{\;n}$ are the predicted density and refractive index from the trained neural network; $\rho_{target}\;,\;\;n_{target}$ are the user-defined target properties; $\omega_{\rho },\;{\;\omega }_{n}$ are the weights for the two objectives; $D_{euclidean}$ is the minimum Euclidean distance between the candidate composition vector x and all compositions in the training dataset; λ is the similarity penalty factor; and ε is a penalty term for constraint violations. The top-ranked compositions are selected as inverse-designed results. This strategy enables efficient exploration of the compositional space while maintaining practical manufacturability. A detailed workflow diagram of the inverse design procedure is provided in Figure S1.

The choice of weighting factors ($\omega_{n}$ = 0.8 for refractive index and $\omega_{\rho }$ = 0.2 for density) is motivated by the following considerations: (i) the refractive index of glass typically requires much higher predictive precision than density, owing to its smaller numerical scale and its strong influence on optical confinement, dispersion, and infrared transmission performance [19,21]; (ii) in practical optical design, refractive index tolerances are significantly tighter than density tolerances. To verify the robustness of the inverse design results with respect to the weighting scheme, we performed a sensitivity analysis by varying the weight ratio $\omega_{n}:\omega_{\rho }$ over the range 0.1:0.9 to 0.9:0.1 in steps of 0.1 and examining the resulting candidate rankings. Results of this sensitivity analysis are presented in Supporting Information Figure S8.

The similarity penalty λ = 0.01 was selected to provide a mild preference for candidate compositions that are close to known glass-forming regions in the training data, without overly constraining the search toward existing compositions. A sensitivity analysis of λ over the range [0, 0.5] is also provided in the Supporting Information Figure S9.

### 2.7. Glass Synthesis and Characterization

Based on the inverse-designed compositions, fluoride glasses were synthesized using a conventional melt-quenching technique. High-purity commercial reagents were employed, including barium fluoride (BaF_2_, Aladdin, ≥99%, Shanghai, China), sodium fluoride (NaF, SCR, ≥99.5%, Shanghai, China), ammonium tetrafluoroaluminate (NH_4_AlF_4_, TY Technology, ≥99.5%, Suzhou, China), magnesium fluoride (MgF_2_, Aladdin, ≥99%, Shanghai, China), and yttrium fluoride (YF_3_, Aladdin, ≥99.9%, Shanghai, China). The raw materials were mixed according to the designed stoichiometric ratios and melted in a platinum crucible at 1050 °C in an electric furnace (ZE Technology, Hangzhou, China). The melt was subsequently cast into a preheated metallic mold at 400 °C and annealed at the same temperature to relieve internal stress, followed by slow cooling to room temperature.

The refractive index at the d-line was measured using a Metricon Model 2010 prism coupler (Metricon Corporation, Pennington, NJ, USA). Prior to measurement, the glass samples were cut, polished, and cleaned using standard procedures to ensure optical-quality surfaces. For each sample, multiple independent measurements were performed, and the averaged value was reported as the final refractive index.

Glass density was determined using the Archimedes buoyancy method. The mass of each dried sample was measured in air and then in deionized water, and the density was calculated accordingly. Each density measurement was repeated three times, and the mean value was used for analysis.

The instrumental uncertainty for refractive index measurements using the Metricon Model 2010 prism coupler is ±3 × 10^−5^. The density measurements performed using an analytical balance (YOKE Instrument, Shanghai, China) carried an uncertainty of ±0.001 g/cm^3^. These uncertainties were incorporated when interpreting deviations between predicted and measured properties.

## 3. Results and Discussion

### 3.1. Prediction Model

The processed dataset was partitioned into training, validation, and testing sets with an 80:10:10 ratio. To refine the high-dimensional feature space, an automated feature selection protocol was implemented to exclude rare components present in fewer than 1% of the samples, thereby focusing the model on the most statistically significant constituents [20,32,33]. Furthermore, a constraint-aware data augmentation scheme—utilizing beta-distribution-based interpolation and additive Gaussian noise—was employed to expand the training diversity while strictly respecting the compositional simplex constraints.

The core GA-BO-BP framework incorporates several advanced structural and optimization enhancements:

- Hybrid Architecture: The network integrates two hidden layers with Batch Normalization and Dropout to ensure numerical stability. A skip connection with a learnable gate was implemented to project input features directly to the output layer, preserving primary compositional signals and facilitating gradient flow [34].
- Robust Objective & Training: To mitigate experimental outliers, Huber loss was employed as the objective function [35]. Training followed a two-stage strategy: an initial Adam phase with Cosine Annealing for global exploration, followed by L-BFGS fine-tuning for high-precision parameter convergence [36].
- Adaptive Regularization: A dynamic $L_{2}$ adjustment scheme monitored the training-to-validation error ratio in real-time, automatically scaling the penalty coefficient to balance model complexity and generalization [37].

Network weights were initialized via GA to bypass unfavorable local minima. Subsequently, BO was utilized to systematically identify the optimal hyperparameter configuration, including hidden layer widths and dropout rates, maximizing the model’s predictive accuracy.

The resulting performance metrics on the held-out test set are summarized in Table 1. The network achieved high prediction accuracy for both density and refractive index, demonstrating strong fitting capability and generalization performance.

**Figure 5:**
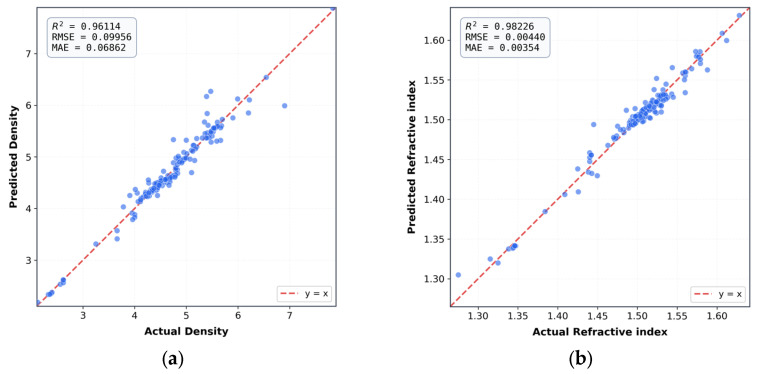
Prediction performance of (**a**) density and (**b**) refractive index, and the top-left corner shows the R^2^ value for each model.

Figure 5 illustrates the prediction performance of the neural network model on the test dataset. As shown in Figure 5a,b, the predicted values of density and refractive index exhibit strong agreement with the corresponding experimental values. This indicates that the model is capable of accurately capturing the underlying composition–property relationships for both target properties. The residual distributions of the density and refractive-index predictions are further shown in Figure S2.

Notably, the refractive index predictions display a tighter clustering around the ideal line and a higher coefficient of determination compared with density, suggesting superior predictive accuracy for refractive index. Overall, these results demonstrate the robustness and generalization capability of the trained model when applied to unseen data.

#### 3.1.1. Cross-Validation Results

**Figure 6:**
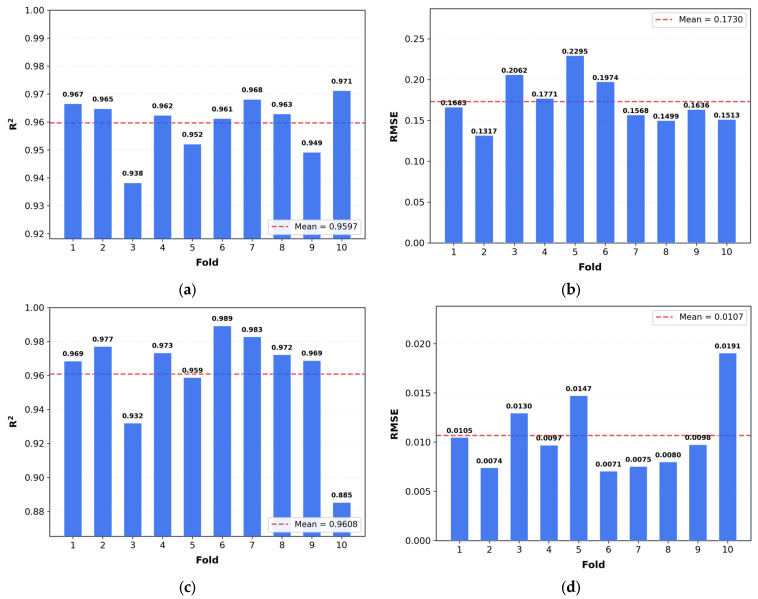
10-fold cross-validation results of the proposed model for density and refractive index prediction: (**a**) fold-wise R^2^ values for density; (**b**) fold-wise RMSE values for density; (**c**) fold-wise R^2^ values for refractive index; and (**d**) fold-wise RMSE values for refractive index.

To assess the robustness of the model beyond a single data partition, 10-fold stratified cross-validation was performed on the training and validation pool (90% of the full dataset), with the fixed test set held out entirely. As shown in Figure 6, the model achieved consistently strong performance for both density and refractive index prediction across different folds.

As shown in Figure 6, the model exhibited consistently strong predictive performance for both density and refractive index across different folds. The mean and standard deviation of the key performance metrics across all 10 folds are summarized in Table 2. For density prediction, the fold-wise R^2^ values ranged from 0.938 to 0.971, with a mean of 0.9597 ± 0.0102, while the corresponding RMSE values varied from 0.1317 to 0.2295, with an average of 0.1730 ± 0.0298. For refractive index prediction, the fold-wise R^2^ values ranged from 0.885 to 0.989, with a mean of 0.9608 ± 0.0308, and the RMSE values ranged from 0.0071 to 0.0191, with an average of 0.0107 ± 0.0039. The low standard deviations across all metrics confirm that the proposed model maintains high predictive accuracy and good stability across different data partitions.

#### 3.1.2. Benchmarking Against Baseline Models

**Figure 7:**
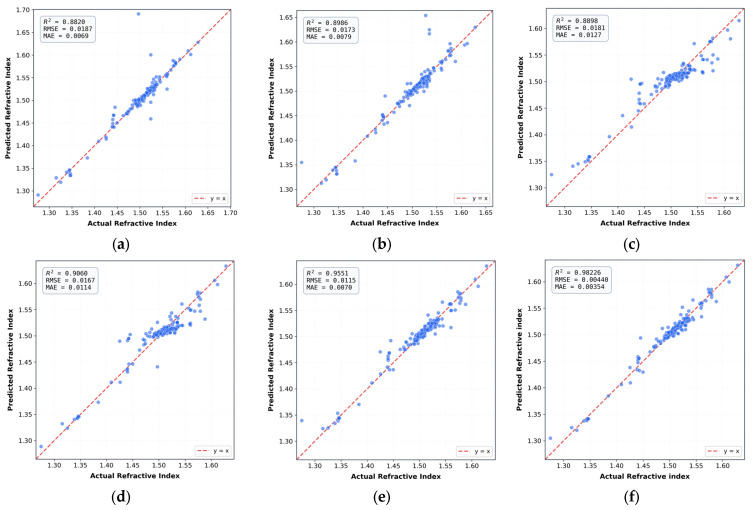
Benchmarking of predictive performance for refractive index across different models: (**a**) SVR; (**b**) RF; (**c**) XGBoost; (**d**) Standard BPNN; (**e**) GA-BPNN with optimized initialization; and (**f**) The proposed GA-BO-BPNN framework. The dashed red line represents the ideal prediction (y = x).

To quantitatively assess the incremental benefits of the proposed GA-BO-BPNN framework, a systematic benchmarking comparison was conducted against widely used machine learning models (SVR, RF, and XGBoost) and ablation variants (Standard BPNN and GA-BPNN with optimized initialization). All models were evaluated on the same held-out test set using R^2^, MAE, and MSE as performance metrics. The predictive performance of these models for refractive index is visualized in Figure 7.

From Figure 7, compared with SVR, RF, and XGBoost, the standard BPNN provides better nonlinear fitting but suffers from variability due to random initialization and manual hyperparameter tuning. Incorporating GA to optimize initial weights (GA-BPNN) improves convergence and prediction stability, with results clustering more closely around the ideal line. The proposed GA-BO-BPNN framework outperforms all baselines, achieving the tightest alignment with experimental values demonstrating superior accuracy, generalization, and robustness. To facilitate direct numerical comparison, the quantitative performance metrics of all models on the held-out test set are summarized in Table 3.

#### 3.1.3. Family-Resolved Error Analysis

To evaluate the model’s generalization across different chemical regimes, prediction errors were disaggregated by glass family: Zr-based (*n* = 368), In-based (*n* = 224), Al-based (*n* = 445), Pb-based (*n* = 174), and Be-based (*n* = 53) systems. Samples were categorized according to whether a given glass-forming fluoride constituted a major component in the composition. Because many fluoride glasses contain more than one major former, these groupings are not mutually exclusive: a single sample may be counted in multiple families. For instance, sample #450 (40InF_3_–40PbF_2_–10ZnF_2_–5BaF_2_–3GaF_3_–2GdF_3_, mol%) is counted under both the In-based and Pb-based families because both InF_3_ and PbF_2_ serve as major components, while sample #224 (28ZrF_4_–25AlF_3_–29PbF_2_–10BaF_2_–5YF_3_–3SrF_2_) simultaneously qualifies as Zr-based, Al-based, and Pb-based owing to the comparable concentrations of all three glass-forming fluorides. This overlap explains why the sum of family-wise sample counts (1264) exceeds the total dataset size (883).

**Figure 8:**
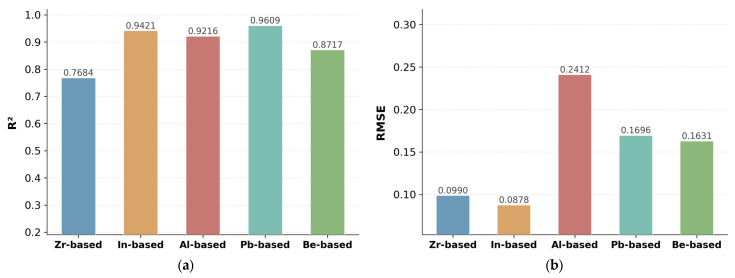

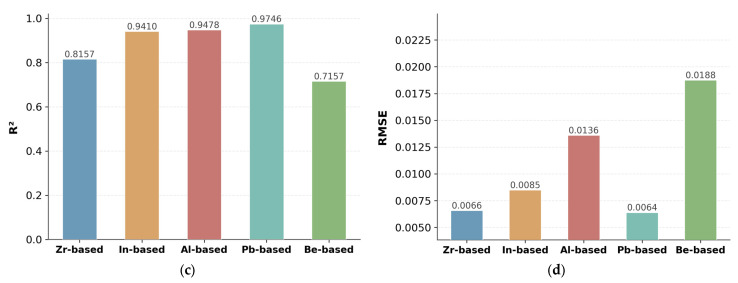
Prediction of the proposed model across different fluoride-glass families: (**a**) R^2^ for density prediction; (**b**) RMSE for density prediction; (**c**) R^2^ for refractive-index prediction; and (**d**) RMSE for refractive-index prediction.

As shown in Figure 8, the model exhibits some differences in predictive performance among these families. For density prediction, the Pb-based family achieves the best overall performance, with an R^2^ of 0.9609, while In-based and Al-based glasses also maintain relatively strong predictive accuracy, with R^2^ values of 0.9421 and 0.9216, respectively. In contrast, the Zr-based and Be-based families show comparatively lower R^2^ values of 0.7684 and 0.8717, indicating reduced predictive consistency in these chemically distinct regimes. A similar trend is observed for refractive index prediction, where Pb-based glasses again show the highest accuracy, followed by Al-based and In-based systems, whereas Zr-based and especially Be-based glasses exhibit noticeably lower performance. The RMSE results further support this trend: while most families remain within a reasonable error range, the Al-based family shows a relatively large density RMSE (0.2412), suggesting greater compositional heterogeneity or a few samples with larger deviations, whereas the Zr-based family exhibits comparatively modest RMSE despite lower R^2^, likely owing to a narrower intrinsic property distribution.

Overall, these results indicate that the proposed framework generalizes well across several major fluoride-glass families, particularly Pb-based, In-based, and Al-based systems, but its performance is less stable for underrepresented or compositionally more challenging families such as Zr-based and Be-based glasses.

### 3.2. Feature Importance

To further interpret the trained neural network model and elucidate the compositional factors governing density and refractive index in fluoride glasses, feature importance analysis was conducted based on SHapley Additive exPlanations (SHAP). SHAP provides a unified, model-agnostic framework to quantify the contribution of each input feature to the model output by attributing prediction deviations from a reference baseline to individual components [38,39,40].

**Figure 9:**
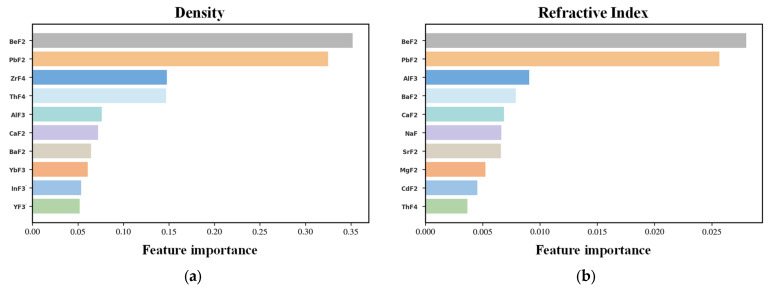
Feature importance for (**a**) density and (**b**) refractive index predictions. Only the top 15 components with the highest importance are shown. Higher values indicate stronger contributions of the corresponding fluoride components to the model output.

Figure 9 summarizes the global feature importance for density and refractive index predictions, ranked by the mean absolute SHAP values. For density prediction (Figure 9a), BeF_2_ and PbF_2_ exhibit the highest mean |SHAP| values, indicating that these two components exert the strongest statistical influence on the model’s density output. Other components such as ZrF_4_, ThF_4_, AlF_3_, and CaF_2_ also contribute noticeably, though to a lesser extent. For refractive index prediction (Figure 9b), PbF_2_ and BeF_2_ again rank highest, followed by AlF_3_, BaF_2_, CaF_2_, and NaF. Notably, the relative ranking of features differs between the two properties, suggesting that the model relies on partially distinct compositional patterns when predicting density versus refractive index.

**Figure 10:**
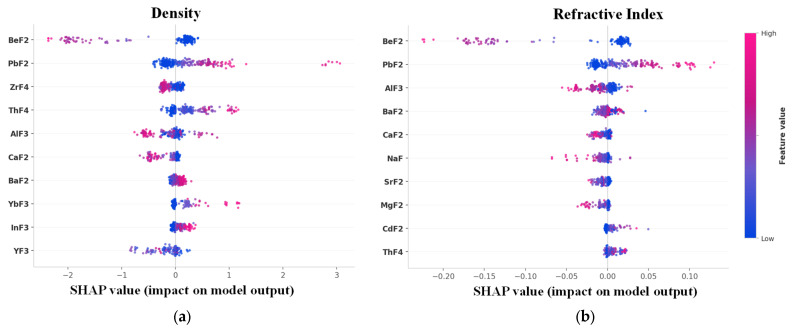
SHAP summary plots obtained from the machine learning prediction models for (**a**) density and (**b**) refractive index predictions. Only the top ten components with the highest correlations are shown. The color scale and horizontal position indicate the direction (positive or negative) and magnitude of each feature’s influence.

To further examine the directionality and nonlinear effects of individual components, SHAP summary plots are shown in Figure 10. Each point represents a sample, with color indicating the corresponding feature value. For density (Figure 10a), higher contents of PbF_2_ and ThF_4_ are predominantly associated with positive SHAP values, whereas lighter fluorides such as BeF_2_ and CaF_2_ tend to be associated with negative SHAP values. For refractive index (Figure 10b), PbF_2_ again shows a strong positive association, whereas BeF_2_ exhibits predominantly negative SHAP values at higher concentrations. AlF_3_ and NaF display mixed positive and negative contributions depending on the compositional context, indicating that the model captures pronounced nonlinear and composition-dependent effects for these features.

To further investigate potential correlation-induced artifacts in the SHAP analysis arising from the closed nature of compositional data, we examined SHAP main effect and SHAP interaction values for the top ten features, with the detailed results provided in the Supporting Information Figures S5–S7.

Overall, the feature-importance analysis confirms that the neural network captures physically meaningful composition–property relationships. Heavy-metal fluorides dominate density prediction, while refractive index depends on a synergistic interplay between heavy-metal fluorides and network-forming components. This interpretability strengthens confidence in the predictive model and provides valuable guidance for inverse design of fluoride glasses with tailored optical properties.

### 3.3. Inverse Design

To evaluate the inverse design capability of the trained model in a proof-of-concept setting, we selected the fluoroaluminate glass system as a case study [41,42,43]. Five components AlF_3_, BaF_2_, NaF, MgF_2_ and YF_3_ were selected to define the composition space, covering both features with relatively high and low importance as indicated by the interpretability analysis. The design goal was to identify combinations with a target refractive index ($n_{d}$ = 1.45) and density (ρ = 4.00 g/cm^3^).

Using the scoring function defined in Section 2.6, we assigned a weight of 0.8 to refractive index and 0.2 to density in the multi-objective optimization. After multiple runs of the inverse-design algorithm, nine candidate glass compositions were obtained. All nine were subsequently synthesized to experimentally assess their glass-forming potential.

However, fluoride glass fabrication is highly sensitive to processing conditions, and not all melts produced homogeneous, bubble-free, and fully amorphous samples. The high volatility of fluoride species at elevated temperatures, severe hygroscopicity that introduces OH^−^ contamination, and the intrinsically narrow glass-forming windows of fluoroaluminate systems collectively increase the risk of crystallization, phase separation, and composition drift [6,44]. As a result, only a subset of the nine compositions exhibited acceptable glass-forming ability. Table 4 presents the compositions and predicted properties for all nine inverse-designed candidate glasses.

Of the nine candidates, Glasses 3, 7, 8, and 9 exhibited varying degrees of crystallization or phase separation during cooling, while Glasses 1, 2 and 6 showed signs of composition drift due to fluoride volatilization at elevated temperatures. Glass 4 and Glass 5 demonstrated the most stable melting behavior, the highest sample quality, and the best reproducibility across repeated trials, yielding homogeneous, bubble-free, and fully amorphous samples. Therefore, they were selected for detailed characterization and comparison with the model predictions in this proof-of-concept study. The compositions and predicted properties of these two glasses are summarized in Table 5.

The measured refractive indices $n_{d}$ of Glass 4 and Glass 5 were 1.43690 and 1.43886, respectively, while their corresponding measured densities were 3.8416 g/cm^3^ and 3.9918 g/cm^3^. Repeated measurements confirmed good consistency: the refractive index values (10 independent measurements per sample) showed a standard deviation of ±1 × 10^−4^, while the densities (5 independent measurements per sample) exhibited a variation of ±0.004 g/cm^3^. A deviation of approximately 0.006 was observed between the measured and predicted refractive indices, while the corresponding deviation in density was 0.031 g/cm^3^ and 0.058 g/cm^3^.

**Figure 11:**
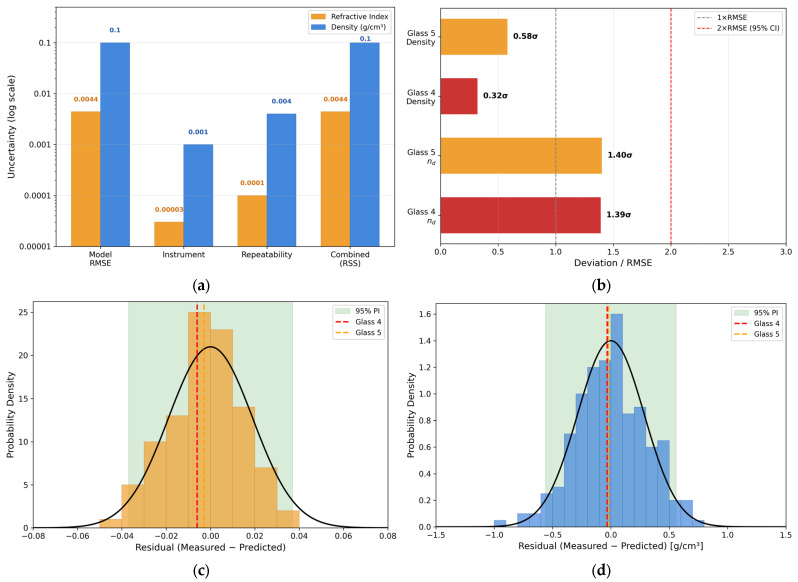
Error Analysis of Glass Samples, (**a**) Uncertainty Budget, (**b**) Normalized Deviations, (**c**) Refractive Index Residual Distribution, (**d**) Density Residual Distribution. In (**c**,**d**), black curves denote the fitted Gaussian probability density function of the residuals, and dashed lines mark the residuals of Glass 4 and Glass 5.

To quantitatively verify whether the measured performance of the inverse-designed glasses falls within the reasonable error range of model predictions, this section conducts a systematic uncertainty analysis from three perspectives: uncertainty budget, normalized deviation analysis, and cross-validation residual distribution, as shown in Figure 11.

Figure 11a presents the combined uncertainty of the model, with deviations from the predicted and measured values attributed to three sources:

1. Model prediction error (RMSE): refractive index of 0.0044 and density of 0.0996 g/cm^3^.
2. Instrument measurement uncertainty: ±3 × 10^−5^ for refractive index and ±0.001 g/cm^3^ for density.
3. Measurement repeatability: standard deviation of ±1 × 10^−4^ for refractive index and ±0.004 g/cm^3^ for density.

The combined uncertainty is calculated using the root sum of squares, resulting in 0.0044 for refractive index and 0.0997 g/cm^3^ for density, with expanded uncertainties of ±0.0088 and ±0.199 g/cm^3^. The model prediction error dominates, with other uncertainties being negligible, indicating that the model’s generalization ability limits prediction accuracy, not measurement precision.

Figure 11b shows that all normalized deviations are within 2σ (RMSE). Density predictions are especially accurate, with Glass 4 and Glass 5 biases under 1 RMSE. Refractive index biases are slightly larger but still within the 95% confidence interval, suggesting the prediction-measurement bias is within expected statistical variation.

Figure 11c,d show that the residuals for Glass 4 and Glass 5 fall within the 28th to 38th percentiles of the residual distribution, far from the 95% prediction interval boundaries. This indicates that the prediction bias is within the model’s typical error range, verifying that the inverse-engineered glass performs consistently with model predictions within the expected uncertainty.

Overall, the synthesized glasses closely matched the target design values for both refractive index and density, while also being consistent with the feature importance trends identified in the model. The proposed framework achieves reliable prediction of density and refractive index. The successful synthesis and characterization of Glass 4 and Glass 5 provide a proof-of-concept demonstration that the inverse design workflow can identify experimentally viable compositions.

### 3.4. Discussion

#### 3.4.1. Analysis of Model Performance Differences

The observed performance advantage of the GA-BO-BPNN framework over XGBoost, RF, and SVR may be attributed to the following factors.

First, composition–property relationships in multi-component fluoride glasses are likely governed by high-order nonlinear interactions among constituents. Neural networks may capture such interactions more naturally through successive nonlinear transformations, whereas tree-based models rely on axis-aligned, piece-wise constant decision boundaries that may be less effective in representing smooth, continuous property variations [45,46]. McElfresh et al. observed that neural networks tend to perform favorably on datasets with smoother target functions, which appears consistent with the physically continuous nature of density and refractive index in glass systems [47].

Second, compositional data reside on a constrained simplex where all components sum to 100 mol%, potentially introducing inter-feature correlations that dense, fully connected layers may handle more flexibly than tree-based methods, which partition each feature axis independently [33]. A similar tendency was reported by Liu et al., who found that deep neural networks may generalize more effectively than RF across high-dimensional compositional spaces [48].

In addition, SVR with an RBF kernel may face scalability challenges in high-dimensional, sparse compositional spaces, where the effectiveness of kernel-based similarity measures could diminish [49].

GA-based initialization and Bayesian hyperparameter optimization likely alleviate the sensitivity to initial conditions and suboptimal hyperparameter selection [46,50], potentially enabling convergence to more favorable regions of the loss landscape. Overall, the obtained predictive performance (refractive index R^2^ = 0.98226, density R^2^ = 0.96114, RMSE = 0.00440 for refractive index and 0.09956 for density) is comparable to or exceeds the performance reported in recent machine learning studies on fluoride glasses. Ahmmad et al. reported R^2^ values of 0.84–0.87 for density prediction of fluoride glasses using artificial neural networks [51], while Fu et al. achieved R^2^ values of 0.94–0.98 for fluorine-containing glasses using RFR, SVR, and XGBR [21].

#### 3.4.2. Physical Interpretation of SHAP Analysis

As shown in Section 3.2, the SHAP analysis identified PbF_2_ and BeF_2_ as the two most statistically influential features for both density and refractive index predictions. For density, higher PbF_2_ and ThF_4_ contents are associated with positive SHAP values, whereas BeF_2_ contributes negatively. For refractive index, PbF_2_ again shows a strong positive association, while BeF_2_ exhibits a predominantly negative influence; AlF_3_, NaF, and BaF_2_ display more complex, mixed directionality depending on the compositional context. These are model-derived statistical patterns; the question addressed below is whether they find support in the known physics and chemistry of fluoride glasses.

From a physicochemical perspective, glass density is primarily governed by the molar mass and ionic radii of the constituent cations. Heavy-metal fluorides such as PbF_2_ (Pb^2+^: 207.2 g/mol) and ThF_4_ (Th^4+^: 232.0 g/mol) are expected to increase density, whereas the light, tetrahedrally coordinated BeF_2_ (Be^2+^: 9.01 g/mol) tends to form open network structures that lower density [5,6]. For refractive index, the Lorentz–Lorenz relation links it to molar refractivity and thus to electronic polarizability [52]. The highly polarizable 6s^2^ lone pair of Pb^2+^ enhances refractivity, whereas the small, low-polarizability Be^2+^ reduces it—trends confirmed experimentally in ZrF_4_-based glasses [53,54]. AlF_3_, BaF_2_, and NaF play dual structural roles (network former vs. modifier, or disrupting connectivity while introducing moderately polarizable cations [42,55]), which can produce competing and non-monotonic effects on both properties.

Comparing these physicochemical expectations with the SHAP results, a notable consistency emerges: the positive SHAP contributions of PbF_2_ and ThF_4_ to density align with their high cationic masses; the negative contributions of BeF_2_ match its low mass and open network tendency; the dominant positive influence of PbF_2_ on refractive index corresponds to the high polarizability of Pb^2+^; and the mixed behavior of AlF_3_, NaF, and BaF_2_ is compatible with their dual structural roles. This alignment suggests that the model’s internal feature weighting is consistent with established glass science, which strengthens confidence in its predictive reliability.

A recent interpretable machine learning study on oxide glasses by Zaki et al. similarly reported that SHAP analysis can reveal such nonlinear, composition-dependent feature contributions that are difficult to capture through simple linear correlation analysis [56]. More recently, Liu et al. applied SHAP interaction analysis to multi-component oxide glasses and confirmed that synergistic effects between components play a critical role in determining optical and physical properties, further supporting the necessity of model-based interpretability approaches for complex glass systems [57].

#### 3.4.3. Impact of Dataset Imbalance on Prediction Performance

The family-resolved error analysis (Section 3.1.3) revealed notable performance disparities across glass families, which may be partially attributed to imbalanced representation in the training dataset. In-based systems achieved relatively high R^2^ values (0.9421 for density), likely benefiting from their extensive coverage in the SciGlass database owing to a long research history in infrared optics. In contrast, Zr-based and Be-based families showed lower accuracy (R^2^ of 0.7684 and 0.8717 for density), which may reflect data scarcity rather than inherent modeling limitations. External validation on an independent literature dataset (Table S2 and Figure S10 in the Supporting Information) further supports this observation. This is a recognized challenge in data-driven materials science, where models tend to develop stronger fitting bias toward majority classes at the expense of underrepresented ones [58].

Zr-based and Be-based system appear to suffer from distinct mechanisms. For Zr-based glasses, although ZrF_4_ is among the most frequently occurring components (Figure 2), the broad compositional diversity within this family—spanning ZBLAN, ZBLA, and numerous higher-order variants—may introduce a wide property distribution that is difficult for a single model to capture uniformly, as reflected by its modest R^2^ despite a relatively moderate RMSE. For Be-based glasses, the lower performance more likely stems from an overall scarcity of training samples, given the limited experimental attention this family has received due to the well-known toxicity of beryllium compounds.

These observations point to several directions for future improvement. First, targeted data collection campaigns focusing on underrepresented families, potentially guided by active learning or uncertainty-based sampling strategies, could help balance the compositional coverage [59]. Second, data augmentation techniques—such as the constraint-aware interpolation employed in this work—could be further refined with family-specific strategies to improve minority-class representation while respecting simplex constraints [60,61]. Third, transfer learning or multi-task learning approaches that leverage shared compositional knowledge across glass families may offer a promising route to improve generalization for data-scarce regimes [62]. Finally, expanding the framework to incorporate additional physical descriptors beyond raw composition—such as ionic radii, electronegativity, or field strength—may enhance the model’s ability to extrapolate across chemically distinct families and further bridge the gap between data-driven prediction and physics-informed glass design.

## 4. Conclusions

In this work, we developed a data-driven computational framework for predicting and inversely designing fluoride glass compositions with targeted refractive index and density. By integrating GA-assisted initialization, Bayesian hyperparameter optimization, and neural-network modeling, the proposed approach achieves reliable property prediction. SHAP-based interpretability analysis reveals that heavy-metal fluorides dominate density variation, while refractive index depends on a more complex interplay among network formers and modifiers. These insights provide physically plausible guidance for composition selection. Two designed compositions were successfully synthesized as high-quality amorphous glasses, with measured properties closely matching the design targets.

Despite the promising performance, several limitations remain. The prediction accuracy varies across glass families, likely due to imbalanced data representation for underrepresented systems, which could be mitigated through targeted data acquisition guided by active learning, improved compositional data augmentation, and the integration of physically meaningful descriptors such as ionic radii and electronegativity. Furthermore, while the preliminary experimental validation of inverse design is encouraging, its general capability can be further improved by incorporating glass-forming ability and thermodynamic stability criteria into the scoring function and integrating physics-informed structural constraints to narrow the search toward experimentally viable compositions.

Overall, this approach provides a promising pathway for accelerating fluoride-glass development in infrared transmitting fibers, laser windows, and mid-IR photonic components by reducing experimental trial-and-error and supporting collaborative design workflows.

## Figures and Tables

**Table 1:** Performance Metrics for the Neural Network Model.

Parameter	Value
Refractive index R^2^	0.98226
Density R^2^	0.96114
Combined R^2^	0.97170
Refractive index (MAE)	0.00354
Density (MAE)	0.06862
Refractive index (RMSE)	0.00440
Density (RMSE)	0.09956

**Table 2:** Summary of 10-fold cross-validation performance metrics.

Metric	Density	Refractive Index
R^2^	0.9597 ± 0.0102	0.9608 ± 0.0308
RMSE	0.1730 ± 0.0298	0.0107 ± 0.0039

**Table 3:** Summary of benchmarking performance metrics for all models on the held-out test set.

Model	Refractive Index			Density		
	R^2^	RMSE	MAE	R^2^	RMSE	MAE
SVR	0.8820	0.0187	0.0069	0.9013	0.1895	0.1148
RF	0.8986	0.0173	0.0079	0.9062	0.1906	0.1016
XGBoost	0.8898	0.0181	0.0127	0.8895	0.2053	0.1279
BPNN	0.9060	0.0167	0.0114	0.8949	0.1695	0.1088
GA-BPNN	0.9551	0.0115	0.0070	0.9453	0.1587	0.1005
GA-BO-BPNN	0.9823	0.0044	0.0035	0.9611	0.0996	0.0686

**Table 4:** Composition (mol%) and predicted properties for all nine inverse-designed fluoride glass candidates.

	BaF_2_	NaF	AlF_3_	MgF_2_	YF_3_	$\mathrm{Predicted}\;\mathrm{Refractive}\;\mathrm{Index}\;(n_{d})$	Predicted Density
Glass 1	31.4	11.8	34.7	5.8	16.3	1.455	4.085
Glass 2	7.2	36.5	44.3	3	9	1.440	3.686
Glass 3	34.2	4.1	19.2	3.2	39.3	1.453	4.220
Glass 4	15.1	15	30.6	6.5	32.8	1.443	3.873
Glass 5	25.8	15.1	26.3	16.8	15.9	1.445	4.050
Glass 6	3.7	37.3	42.5	6.7	9.8	1.448	3.643
Glass 7	38.5	6.2	16.5	5.2	33.6	1.465	4.160
Glass 8	39	1.6	16.1	10.4	32.9	1.460	4.180
Glass 9	15.2	16.7	51.5	14.6	2	1.439	3.884

**Table 5:** Composition (mol%), target properties, and predicted properties of the two fluoride glasses synthesized in this study. Closest glass represents the composition in the training dataset most similar to the candidate, based on minimal Euclidean distance.

Fluoride	Glass 4	Glass 5	Closest Glass
BaF_2_	15.1	25.8	20.0
NaF	15.0	15.1	5.0
AlF_3_	30.6	26.3	30.0
MgF_2_	6.5	16.8	13.0
YF_3_	32.8	15.9	30.0
CaF_2_	0	0	2.0
Target property	Glass 4	Glass 5	
Refractive index ($n_{d}$)	1.45	1.45	
Density	4.00	4.00	
Predicted property	Glass 4	Glass 5	
Refractive index ($n_{d}$)	1.443	1.445	
Density	3.873	4.050	

## Data Availability

The original contributions presented in this study are included in the article/Supplementary Material. Further inquiries can be directed to the corresponding authors.

## Supplementary Materials

The following supporting information can be downloaded at: https://www.mdpi.com/article/10.3390/ma19091685/s1, Figure S1: Detailed workflow of the inverse design procedure; Figure S2: Residual distribution of the neural network model predictions for (a) density and (b) refractive index; Figure S3: Pearson correlation heatmap of all compositional features used in model training; Figure S4: Pearson correlation matrix of the top ten compositional features ranked by global feature importance, together with density and refractive index; Figure S5: SHAP main effect of the top ten input features in density predictions; Figure S6: SHAP main effect of the top ten input features in refractive index predictions; Figure S7: SHAP interaction values for the top ten features for (a) density and (b) refractive index; Figure S8: Sensitivity of inverse design results to the objective weight ratio; Figure S9: Effect of similarity penalty weight (λ) on inverse design results; Figure S10: Predicted versus experimental values for (a) density and (b) refractive index on the external validation dataset; Table S1: Summary of key training logs and configuration parameters for the GA–BP–BO framework; Table S2: Compositions and experimentally measured densities and refractive indices of fluoride glass samples collected from literature for external validation.

## Abbreviations

The following abbreviations are used in this manuscript:

BPNN	Backpropagation Neural Network
GA	genetic algorithms
BO	Bayesian optimization
SHAP	SHapley Additive exPlanations
