# Supplementary material for: Data-Driven Prediction and Inverse Design of Fluoride Glasses via Explainable GA-BP Neural Networks
Source: Materials (Basel). 2026 Apr 22;19(9):1685. doi: 10.3390/ma19091685 (PMC13165318; doi:10.3390/ma19091685)
Supplement: Supplementary file 1 [file materials-19-01685-s001.zip › materials-4231878-supplementary.pdf]

Supporting Information

S.1. Backpropagation Neural Network

The feedforward neural network consists of an input layer, two hidden layers, and an output layer. During forward propagation, the input to the j-th neuron in the l-th layer is given by:

$$z_j^{(l)} = \sum_i w_{ji}^{(l)} a_i^{(l-1)} + b_j^{(l)}$$

where $w_{ji}^{(l)}$ is the weight connecting the i-th neuron in the l-1-th layer to the j-th neuron in the l-th layer, $a_i^{(l-1)}$ is the output of the previous layer, and $b_j^{(l)}$ is the bias term. The neuron output is obtained through an activation function:

$$a_j^{(l)} = f\left(z_j^{(l)}\right)$$

During backpropagation, the prediction error is propagated backward to compute the gradient of the loss function with respect to the network parameters. To mitigate the influence of experimental outliers, the Huber (Smooth L1) loss with $\beta = 0.05$ is adopted as the objective function instead of the conventional mean squared error. The error term for each neuron is calculated using the chain rule:

$$\delta_j^{(l)} = f^{'\left(z_j^{(l)}\right)} \sum_k \delta_k^{(l+1)} w_{kj}^{(l+1)}$$

where $f^{'}$ is the derivative of the activation function, and $\delta_k^{(l+1)}$ is the error term of the k-th neuron in layer l+1. Weights and biases are updated accordingly:

$$w_{ji}^{(l)} \leftarrow w_{ji}^{(l)} - \eta \cdot \delta_j^{(l)} \cdot a_i^{(l-1)} b_j^{(l)}$$

where η is the learning rate.

S.2. Genetic Algorithm for Network Parameter Optimization

The genetic algorithm was used to optimize the initial weights and biases of the BP neural network. Each chromosome encodes a candidate parameter set using real-valued representation.

The GA procedure consists of population initialization, fitness evaluation, selection, crossover, and mutation. Tournament selection and multi-point crossover were adopted. A dynamic mutation strategy was applied, defined as:

$$x_{new} = x + (ub - x) \cdot fg$$

$$x_{new} = x - fg \cdot (x - lb)$$

where fg is a dynamically adjusted mutation factor, and *ub* and *lb* are upper and lower bounds of each gene, respectively.

S.3. Bayesian Optimization for Hyperparameter Selection

To efficiently determine optimal hyperparameters for the neural network, Bayesian optimization was employed. BO constructs a surrogate model, to describe uncertainty in hyperparameters relative to model performance:

$$f(x) \sim GP(\mu(x), k(x, x'))$$

where $\mu(x)$ is the mean function, and $k(x, x')$ is the covariance function describing correlations between hyperparameters.

BO iteratively updates the GP model based on evaluated points and maximizes an acquisition function, such as Expected Improvement (EI):

$$EI(x) = \mathbb{E}[\max(f_{min} - f(x), 0)]$$

where f_{min} is the minimum observed value, and $f(x)$ is the predicted value at a candidate point.

In this work, Bayesian optimization was implemented using the Optuna framework with a Tree-structured Parzen Estimator (TPE) sampler to efficiently navigate the hyperparameter space. A total of 50 trials were conducted, with a MedianPruner applied to terminate unpromising configurations early and a timeout of 600 s imposed to limit computational cost. The search space covered hidden layer 1 neurons (40–200), hidden layer 2 neurons (20–100), dropout rate (0.0–0.3), learning rate (1×10^{-5}–5×10^{-2}, log scale), and training epochs (150–800, step size of 20). By coupling BO with GA-based weight initialization and BP training, the proposed framework achieves stable convergence and improved generalization performance.

S.4. Inverse Design and Scoring Function

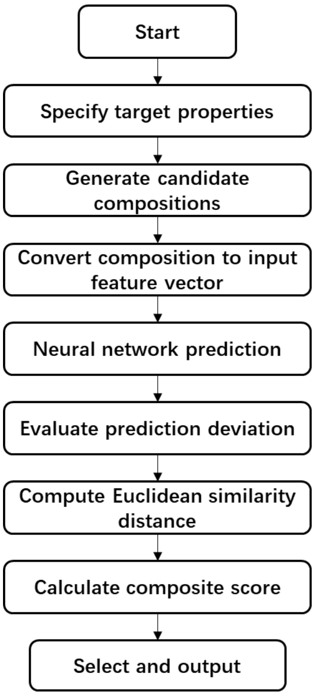

Fig. S1. Detailed workflow of the inverse design procedure.

The Euclidean distance was computed across the full set of normalized compositional features, with the dimensionality determined by the training dataset.

$$D_{euclidean} = \min_j \left(\sqrt{\sum_{i=1}^{n} \left(x_i - x_{i,j} \right)^2} \right)$$

where n denotes the dimensionality of the compositional feature vector, corresponding to the number of normalized components present in the training dataset. x_i is the candidate composition and x_j is the j-th sample in the training dataset.

S.5. Definitions of performance metrics

The Pearson correlation coefficient (R) is defined as:

$$R = \frac{\sum_{i=1}^{N} (y_i - \bar{y})(\hat{y}_i - \bar{\hat{y}})}{\sqrt{\sum_{i=1}^{N} (y_i - \bar{y})^2} \sqrt{\sum_{i=1}^{N} (\hat{y}_i - \bar{\hat{y}})^2}}$$

The coefficient of determination (R^2) is expressed as:

$$R^2 = 1 - \frac{\sum_{i=1}^{N} (y_i - \hat{y}_i)^2}{\sum_{i=1}^{N} (y_i - \bar{y}_i)^2}$$

The root mean square error (RMSE) is defined as:

$$RMSE = \sqrt{\frac{1}{N}\sum_{i=1}^{N}(y_i - \hat{y}_i)^2} \quad (1)$$

The mean absolute error (MAE) is given by:

$$MAE = \frac{1}{N}\sum_{i=1}^{N}\left|y_i - \hat{y}_i\right| \quad (2)$$

where y_i and $\hat{y}_i$ denote the experimental and predicted values, respectively, $\bar{y}$ and $\bar{\hat{y}}$ represent their mean values, and N is the number of samples.

S.6. Training Log

The main runtime settings and training logs of the GA–BP–BO framework are summarized in Table S1 for reproducibility.

Table S1. Summary of key training logs and configuration parameters for the GA–BP–BO framework.

Parameter	Description	Default	Notes
**Data**			
train / val / test ratio	Dataset split ratios	0.80 / 0.10 / 0.10	Stratified by density
min_nonzero_ratio	Feature selection threshold	0.01	Drop rare components
input_scaler	Input normalization	MinMax [0,1]	Refit per CV fold
output_scaler	Output normalization	MinMax [0,1]	
**Data Augmentation**			
num_new_samples	Augmented samples to generate	500	Beta(2,2) interpolation
input_noise	Gaussian noise on inputs	0.015	Std dev
output_noise	Gaussian noise on outputs	0.003	Std dev
**Neural Network Architecture**			
hidden1	Hidden layer 1 neurons	BO-optimized	Range: 40–200
hidden2	Hidden layer 2 neurons	BO-optimized	Range: 20–100
dropout	Dropout probability	BO-optimized	Range: 0.0–0.3
activation	Activation function	Tanh	Both hidden layers

use_batchnorm	Batch normalization	True	Before activation
skip_gate	Residual skip connection gate	0.1	Learnable parameter
weight_init	Weight initialization	Xavier Uniform	
**Genetic Algorithm (Weight Initialization)**			
population_size	Population size	20	
max_generations	Number of generations	20	
crossover_prob	Crossover probability	0.85	Two-point crossover
mutation_prob	Mutation probability	0.15	Cosine-annealed strength
weight_bound	Weight search bounds	±1.5	
elite_count	Elite preservation count	3	Tournament selection (k=3)
**Bayesian Optimization (Hyperparameters)**			
n_trials	Number of Optuna trials	50	TPE sampler
lr_range	Learning rate search range	1e-5 – 5e-2	Log scale
epochs_range	Epochs search range	150 – 800	Step = 20
timeout	Optimization timeout	600 s	MedianPruner
**Training — Stage 1: Adam**			
max_epochs	Maximum training epochs	300	
patience	Early stopping patience	30	Monitors val MSE
loss_function	Loss function	SmoothL1 (Huber)	$\beta = 0.05$
grad_clip	Gradient clipping norm	5.0	
l2_lambda_init	Initial L2 regularization	0.008	Dynamic: [1e-6, 0.1]
l2_increase_factor	L2 increase factor	1.05	When overfitting detected
l2_decrease_factor	L2 decrease factor	0.95	When both losses improve

scheduler	LR scheduler	CosineAnnealingWR	T_0=50, T_mult=2
n_restarts	Random restart count	5	Best model selected
**Training — Stage 2: L-BFGS Fine-tuning**			
lr	L-BFGS learning rate	0.5	
max_steps	Maximum outer steps	50	Early stop patience = 10
max_iter	Inner iterations per step	20	
line_search_fn	Line search method	strong_wolfe	
**Cross-Validation**			
n_folds	Number of folds	10	Stratified K-Fold
mode	CV purpose	Evaluation only	Final model retrained on all data
**Inverse Design**			
num_candidates	Random candidate compositions	100,000	Dirichlet(α=1)
top_k	Top results returned	10	
density_weight	Density error weight	0.2	In scoring function
ri_weight	Refractive index error weight	0.8	In scoring function
similarity_penalty	Similarity penalty coefficient	0.01	Euclidean to nearest train sample
**SHAP Interpretability Analysis**			
explainer	SHAP explainer type	KernelExplainer	
n_background	Background samples	min(100, N)	shap.sample()
nsamples	SHAP perturbation samples	128	

S.7. Residual distribution analysis

Figure S2 shows the probability distributions of the residuals from the neural network model predictions. The histograms of density and refractive index residuals are both centered around zero and display symmetric, unimodal distributions, indicating the absence of systematic prediction bias. The relatively narrow spread of the residuals suggests that prediction errors are generally small. Compared with density, the refractive index residuals are more tightly clustered, corresponding to lower prediction variance.

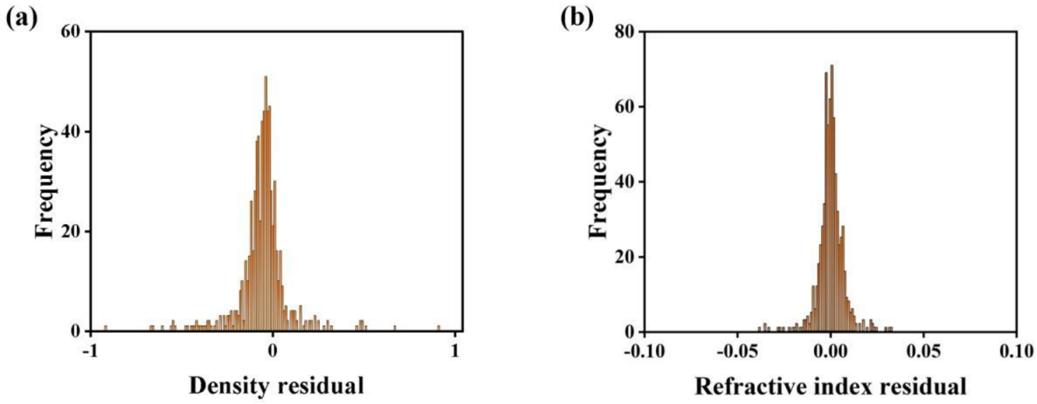

Fig. S2. Residual distribution of the neural network model predictions for (a) density and (b) refractive index.

S.8. Feature importance and correlation analysis

To further examine the statistical relationships among input features and assess potential redundancy, feature correlation analysis was performed on the full compositional dataset. Figure S3 shows the pairwise Pearson correlation coefficients for all compositional features used in model training. Overall, most feature pairs exhibit weak correlations, with correlation coefficients concentrated near zero, indicating a low degree of linear dependence across the high-dimensional compositional space. Several localized regions of moderate correlation can be observed, which are mainly associated with chemically related fluoride components or compositional constraints arising from normalization of molar fractions. Importantly, no large blocks of strong correlation are present, suggesting that the input feature set does not suffer from severe multicollinearity. This supports the stability of the trained neural network and reduces the risk of biased feature attribution.

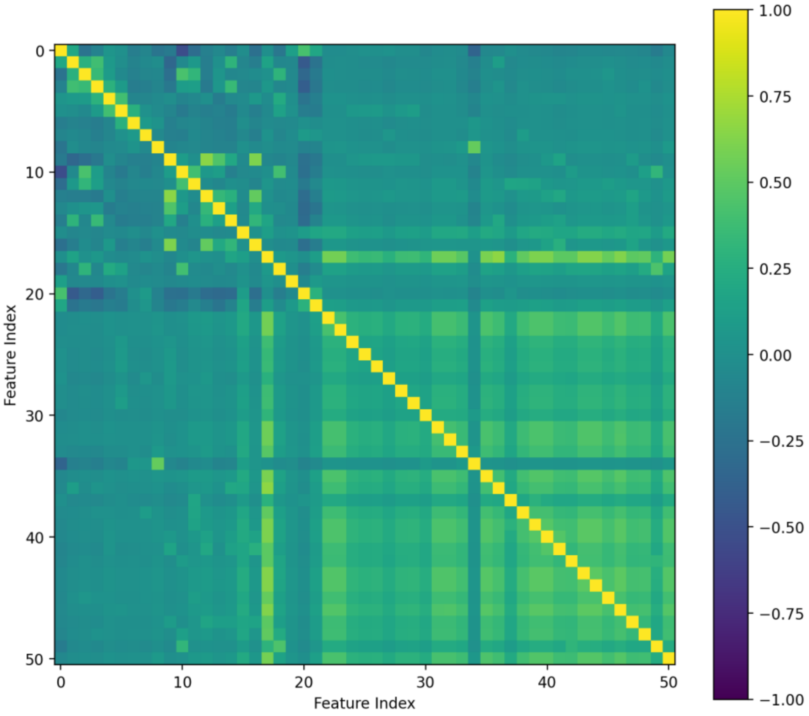

Fig. S3. Pearson correlation heatmap of all compositional features used in model training. The color scale indicates the correlation coefficient.

The Pearson correlation matrix of the top ten compositional features, together with density and refractive index, is shown in Figure S4. The dominant features exhibit weak to moderate mutual correlations, indicating limited linear multicollinearity. While density and refractive index are strongly correlated, the compositional features display distinct correlation patterns with each property, supporting the robustness and interpretability of the model.

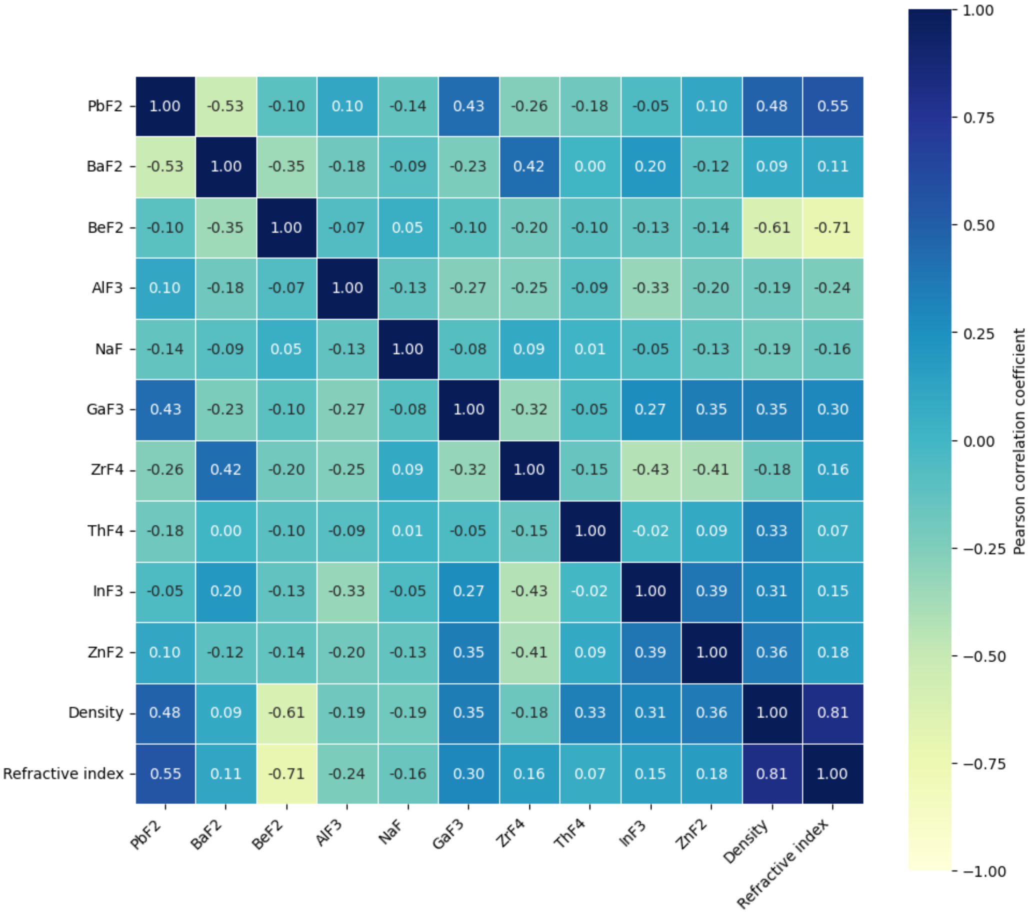

Fig. S4. Pearson correlation matrix of the top ten compositional features ranked by global feature importance, together with density and refractive index.

S.9. Further SHAP Analysis

SHAP main effect plots reveal the marginal effect of a single component as its concentration varies—specifically, the direction and magnitude by which a given feature shifts the model prediction at different compositional levels. Accordingly, the top 10 most influential components identified from the feature importance analysis for refractive index and density were selected, and their SHAP main effect plots were constructed to visually characterize the nonlinear mapping relationships between key compositional variables and the target properties.

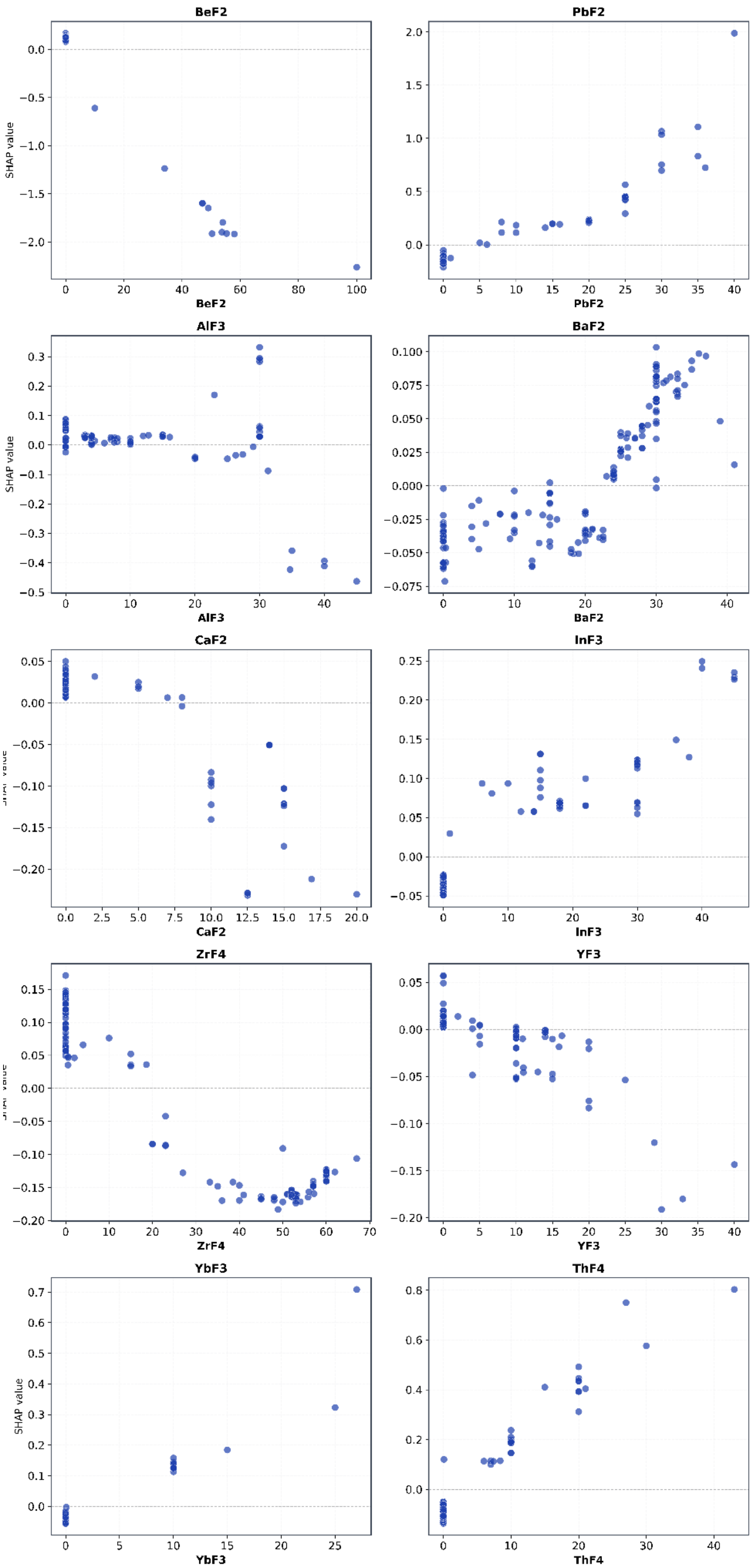

Fig. S5. SHAP main effect of top-10 input feature in density predictions.

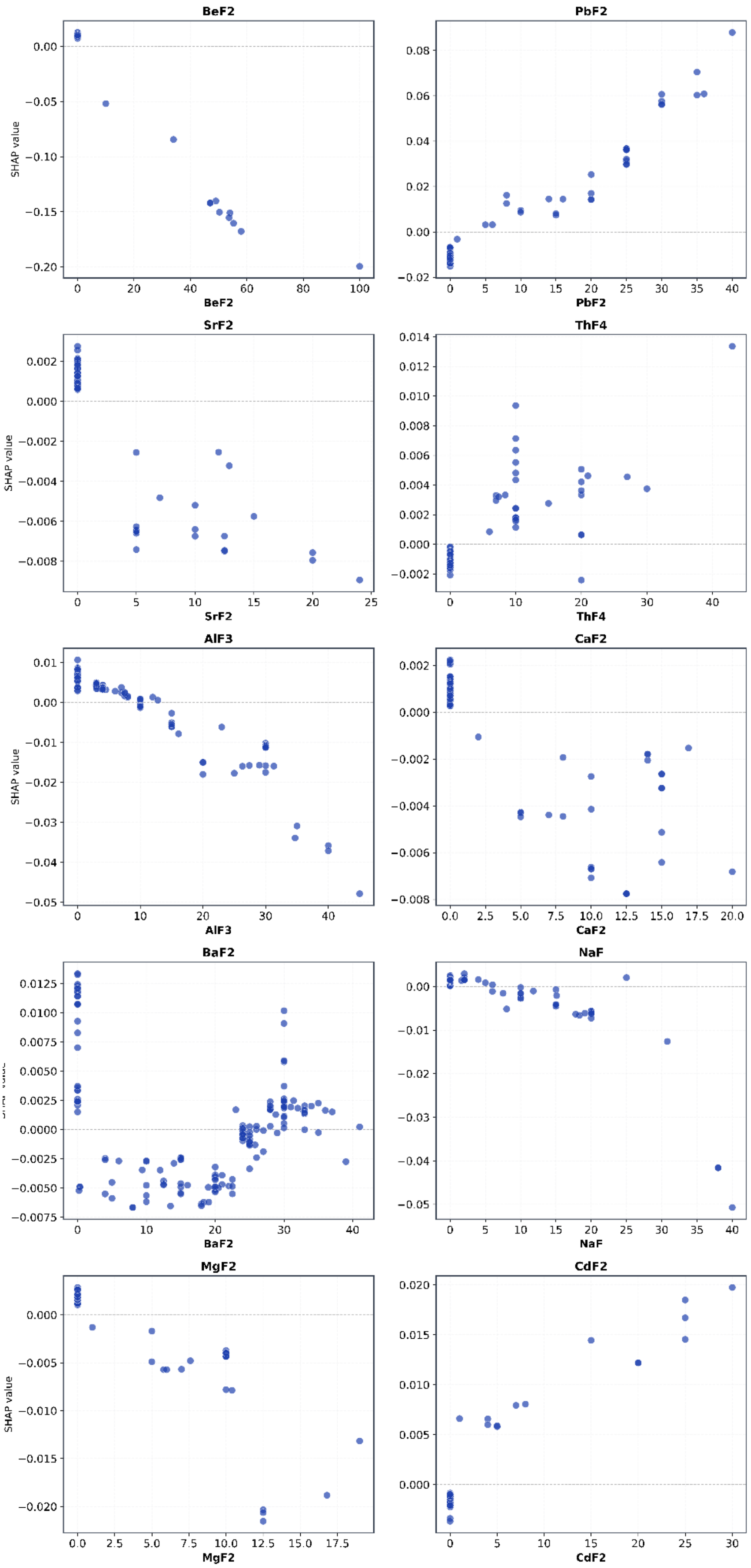

Fig. S6. SHAP main effect of top-10 input feature in refractive index predictions.

As shown in Fig. S5 and Fig. S6, the SHAP main effect plots isolate the marginal contribution of each individual component to the predicted density and refractive index, respectively. Unlike SHAP interaction values or total SHAP attributions, the main effect captures only the direct, first-order influence of a single feature on the model output, and is therefore largely insensitive to correlations among compositional variables in the dataset. This makes it a particularly suitable interpretability tool for compositional data, where inter-feature dependencies are inherent. Across both properties, BeF_2 and PbF_2 emerge as the two most dominant components acting in opposite directions: BeF_2 consistently exerts a strong negative effect, while PbF_2 drives both properties upward. Several components such as AlF_3 and ZrF_4 exhibit nonlinear SHAP profiles with clear transitional thresholds, suggesting composition-dependent structural reorganization. Overall, the main effect analysis provides a robust, correlation-resistant view of individual composition–property relationships and identifies critical compositional thresholds that can inform targeted fluoride glass design.

To investigate potential correlation-induced artifacts in the SHAP analysis arising from the closed nature of compositional data, SHAP interaction values were computed for the top ten features identified by the global importance ranking. The interaction analysis reveals the extent to which the SHAP value of one feature depends on the value of another feature.

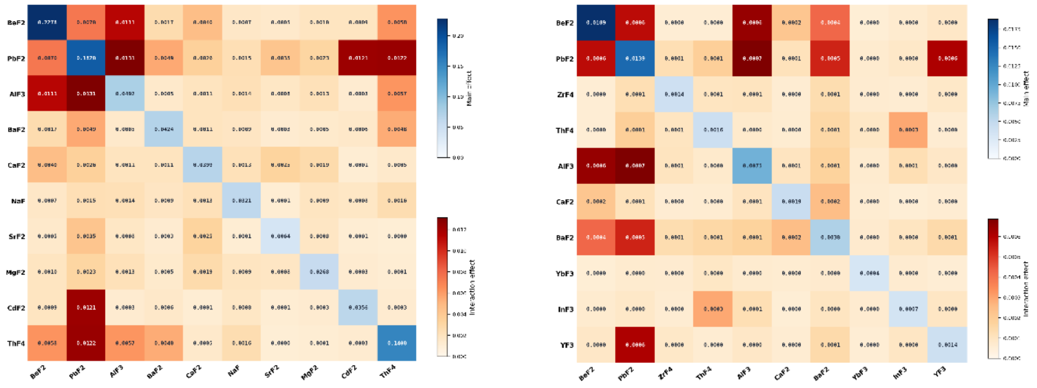

(**a**) (**b**)

Fig. S7. SHAP interaction values for the top ten features for (a) density and (b) refractive index prediction. Off-diagonal entries represent pairwise interaction effects; diagonal entries represent the main effects.

For both target properties, diagonal main effects exceed off-diagonal interaction terms by approximately one to two orders of magnitude, confirming that each component's contribution to the model prediction is predominantly additive and independent. In the density model (Fig. S7a), BeF_2 exhibits the largest main effect (0.2278), followed by PbF_2 (0.1820) and AlF_3 (0.0492), consistent with the global importance hierarchy established in Section 3.2. The most pronounced pairwise interactions involve PbF_2–AlF_3 (0.0131), PbF_2–CdF_2 (0.0121), and PbF_2–ThF_4 (0.0122), which may reflect genuine physicochemical coupling among heavy-metal fluorides in modifying the glass network packing efficiency. For the refractive index model (Fig. S7b), BeF_2 (0.0189) and PbF_2 (0.0178) again dominate the main effects, while all pairwise interaction terms remain uniformly below 0.005, indicating minimal inter-feature dependence.

These findings provide two important conclusions. First, the compositional closure constraint does not introduce significant artificial interactions into the SHAP analysis,

thereby validating the reliability of the feature importance rankings reported in the main text. Second, the small but non-negligible interactions observed in the density model—particularly those involving PbF_2—suggest potential synergistic compositional effects on glass density that merit further physicochemical investigation.

S.10. Sensitivity Analysis of Objective Weights

To evaluate the robustness of the inverse design results with respect to the weighting scheme, we repeated the inverse design procedure with weight ratios (ω_ρ: ω_n) ranging from 0.9:0.1 to 0.1:0.9, corresponding to ω_n values from 0.1 to 0.9 in steps of 0.1. For each weight configuration, the same candidate generation and scoring protocol described in Section 2.6 was applied, and both the average score of the top-10 ranked candidates and the composition of the top-1 candidate were recorded.

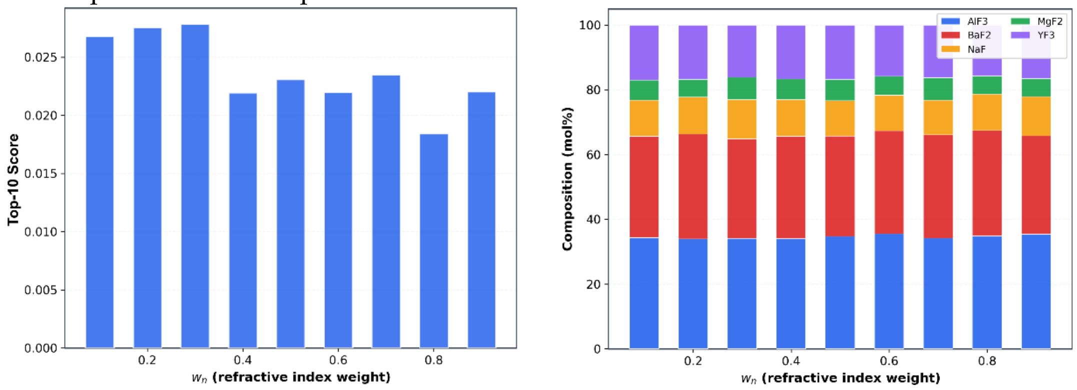

(a) (b)

Fig. S8. Sensitivity of inverse design results to the objective weight ratio (ω_ρ: ω_n): (a) mean Top-10 score and (b) Top-1 candidate composition as a function of ω_n.

As shown in Fig. S8a, the mean Top-10 score remains within a narrow band (approximately 0.019–0.030) across all weight ratios, indicating that the scoring landscape is not dominated by any single weighting configuration. A modest increase in the average score is observed at lower ω_n values (0.1–0.3), where the density objective receives greater emphasis; this likely reflects the comparatively larger numerical scale of density deviations relative to refractive index deviations. As ω_n increases beyond 0.4, the scores stabilize at a slightly lower level (approximately 0.019–0.023), suggesting that higher refractive-index weights impose a tighter constraint that effectively narrows the set of high-scoring candidates.

Fig. S8b presents the composition of the top-1 candidate at each weight ratio. Notably, the optimized compositions remain broadly consistent across the entire ω_n range: AlF_3 consistently constitutes the largest fraction (approximately 26–35 mol%), followed by BaF_2 (approximately 15–26 mol%), YF_3 (approximately 14–20 mol%), NaF (approximately 5–15 mol%), and MgF_2 (approximately 5–17 mol%). Although minor compositional shifts are observed—for instance, the BaF_2 fraction tends to increase slightly at higher ω_n values while the NaF fraction decreases—the overall compositional profile of the

top-ranked candidate exhibits no abrupt transitions or qualitative changes across the weight spectrum.

These results collectively demonstrate that the inverse design framework is robust with respect to the choice of objective weights. The selected weight ratio of ω_n = 0.8 and ω_ρ = 0.2 used in the main text falls within a stable performance region and does not represent an outlier configuration.

S.11. Sensitivity Analysis of Similarity Penalty Weight

To evaluate the effect of the similarity penalty weight (λ) on inverse design performance, a sensitivity analysis was conducted by varying λ from 0 to 0.5 with fixed property weights.

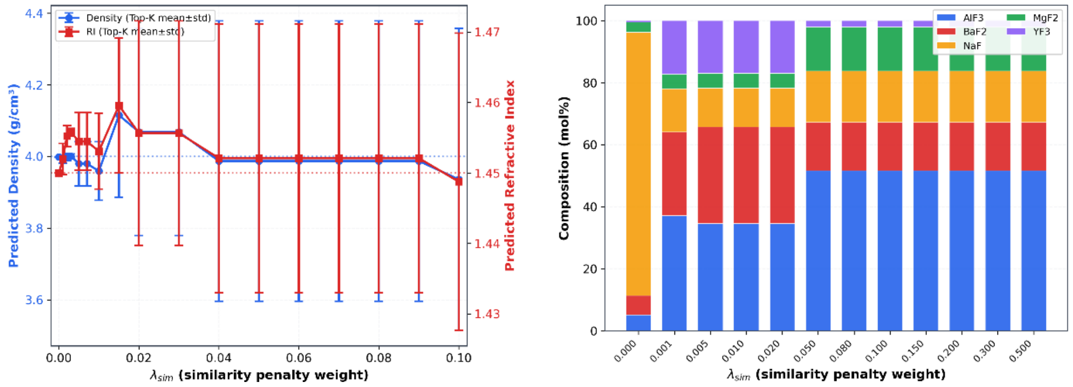

(**a**) (**b**)

Fig. S9. Effect of similarity penalty weight (λ) on inverse design results: (a) predicted density and refractive index of Top-10 candidates as a function of λ, with dashed lines indicating target values; (b) Top-1 composition distribution across the five selected components at different λ values.

As shown in Fig. S9a, when $\lambda \leq 0.02$, the predicted density and refractive index of the Top-K candidates remain close to the target value. A sharp transition occurs around $\lambda \approx 0.03$-0.05, where the predicted density drops to 3.6-4.4 g/cm^3 and the refractive index decreases to 1.43-1.47, indicating that the similarity penalty begins to dominate and forces the optimizer toward well-sampled but off-target regions. Fig. S9b corroborates this finding: at low λ, the compositions are well-distributed among all five components, whereas at high λ, the compositions collapse toward AlF_3 and BaF_2 dominance, significantly reducing compositional diversity.

Accordingly, λ = 0.01 was adopted for subsequent inverse design. At this value, the property deviations remain small while the nearest training sample distance is reduced from 59 (at λ = 0) to 1.5, ensuring that the designed compositions reside within a well-modeled region of the feature space without sacrificing property accuracy.

S.12. External Validation Using Independent Literature Data

To evaluate the generalization capability of the trained model beyond the original training dataset, an external validation study was conducted using fluoride glass compositions and properties reported in the recent literature that were not included in the dataset. This independent assessment provides a stringent test of the model's ability to extrapolate to previously unseen compositional regions.

A total of 11 fluoride glass samples were collected from an independent publication *[Poulain, Marcel. "Heavy metal fluoride glasses: A tutorial review." APL Photonics 9.9 (2024). https://doi.org/10.1063/5.0226668]*, covering a range of glass-forming systems.

Table S2. Compositions and experimentally measured densities and refractive indices of fluoride glass samples collected from literature for external validation.

ZrF_4	HfF_4	BaF_2	LaF_3	AlF_3	NaF	YF_3	SrF_2	CaF_2	ZnF_2	ThF_4	InF_3	CdF_2	Density	Refractive indices
57		34	5	3									4.54	1.519
	57	36	3	4									5.88	1.504
52		24		4	20								4.28	1.497
53		20	4	3	20								4.52	1.498
		20			20		20				40		5.14	1.5005
		15			20		20	5			40		5.1	1.498
		15			30		20				30	5	4.96	1.487
		20		40		20		20					4	1.44
				28.75	22.6	28.7				20			5.1	1.487
		30		20	20	20			10				4.49	1.488
20		20		20		10		20	10				4.32	1.486

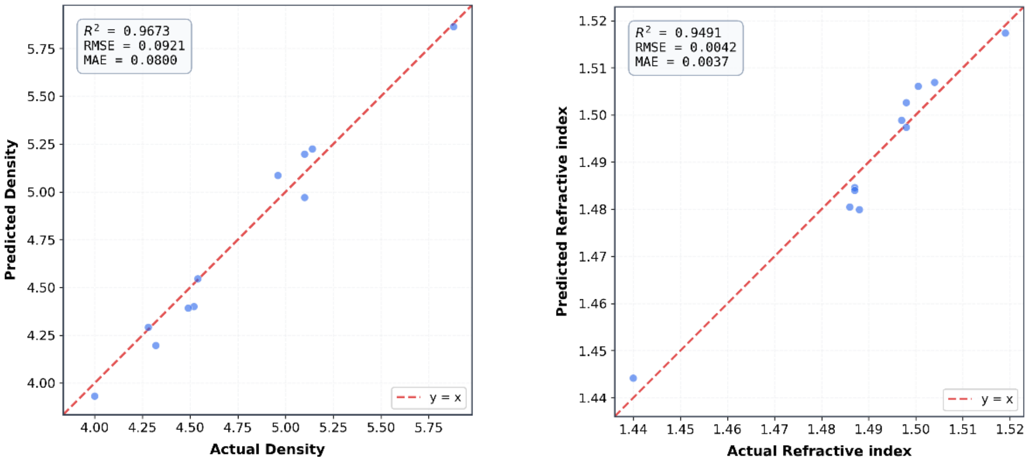

(a) (b)

Fig. S10. Predicted versus experimental values for (a) density and (b) refractive index on the external

validation dataset.

The external validation results are presented in Table S2 and Fig. S10 The model achieved R^2 values of 0.9673 and 0.9491 for density and refractive index, respectively, with corresponding RMSE values of 0.0921 g/cm^3 and 0.0042. Although the R^2 values are lower than those obtained on the internal test set, this is primarily attributed to the limited number of samples in the external validation set. With a small sample size, a few data points with relatively large deviations exert a disproportionate influence on the R^2 statistic, causing it to decrease noticeably. The low MAE values further confirm that the overall prediction errors remain practically small. These results demonstrate that the model possesses satisfactory generalization capability for fluoride glass property prediction across diverse databases.
